# Relative frequency of hepatitis B virus, human papilloma virus, Epstein-Barr virus, and herpes simplex viruses in the semen of fertile and infertile men in Shiraz, Iran: A cross-sectional study

**DOI:** 10.18502/ijrm.v19i8.9617

**Published:** 2021-09-09

**Authors:** Hossein Afrakhteh, Negar Joharinia, Akhtar Momen, Razieh Dowran, Abouzar Babaei, Parisa Namdari, Mohammad Motamedifar, Bahia Namavar Jahromi, Jamal Sarvari

**Affiliations:** ^1^Department of Bacteriology and Virology, Shiraz University of Medical Sciences, Shiraz, Iran.; ^2^Shiraz HIV/AIDS Research Center, Institute of Health and Department of Bacteriology and Virology, School of Medicine, Shiraz University of Medical Sciences, Shiraz, Iran.; ^3^Department of Obstetrics and Gynecology, School of Medicine, Shiraz University of Medical Sciences, Shiraz, Iran.; ^4^Gastroenterohepatology Research Center, Shiraz University of Medical Sciences, Shiraz, Iran.

**Keywords:** Male infertility, Hepatitis B virus, Human papilloma virus, Epstein-Barr virus, Herpes simplex viruses.

## Abstract

**Background:**

About 8-12% of couples on reproductive age suffers from infertility worldwide. Since 1993, the role of genital tract infections by microbes, including viruses that can infect the sperm, in human infertility has been proposed.

**Objective:**

To investigate the frequency of hepatitis B virus (HBV), human papilloma virus (HPV), Epstein-Barr virus (EBV), and herpes simplex virus (HSV) infection in the semen of fertile and infertile men referred to the Mother and Child Hospital, Shiraz, Iran.

**Materials and Methods:**

In this cross-sectional study, 350 men including 200 infertile and 150 fertile men were included. All semen samples were allowed to liquefy, followed by the assessment of sperm parameters. DNA was extracted using a DNA extraction kit (CinaGene, Tehran, Iran) according to the manufacturer's instructions. Detection of HBV, HPV, EBV, and HSV1/2 was done by the PCR method.

**Results:**

The mean age of the participants was 36 ± 7 yr. Molecular results showed that 16 samples (8%) of infertile men and 5 (3.3%) of fertile men were positive for HBV, which was not statistically significant (p = 0.069). Only one sample of the fertile participants was positive for HPV. None of the semen samples of the infertile or fertile groups was positive for the presence of EBV or HSV1/2.

**Conclusion:**

The results of this study indicated that HBV, HPV, EBV, and HSV might not be involved in men's infertility. Further studies are recommended for clarifying the role of these viruses in infertility.

## 1. Introduction

Inability to conceive after 12 months of unprotected and regular sexual intercourse deﬁned as infertility. It has been reported that around 8-12% of couples suffer from infertility worldwide (1), and overall 50% of infertile cases are associated with men (2). The most common condition related with male infertility include varicocele, endocrine disorders, spermatic duct obstruction, anti-sperm antibodies, gonadotoxins, drugs, cryptorchidism, infection, sexual dysfunction, and ejaculatory failure (3). Viruses could infect the genital tract and impair the semen by various mechanisms (4, 5). Several viruses, such as cytomegalovirus (CMV), Epstein-Barr virus (EBV), human papillomavirus (HPV), hepatitis B virus (HBV), herpes simplex virus (HSV), and human immunodeficiency virus, have been detected in the semen of asymptomatic men and might be involved in male infertility (4, 6).

HBV is a member of the Hepadenaviridae family, which is transmitted by infected blood and semen (7). HBV DNA has been found in the semen of HBV-infected patients, but not in HBV-negative patients (4). It has been reported that HBV infection has been linked with low quality of sperm resulting increase the frequency of infertility in men (8, 9). Su and colleagues stated that the risk of infertility is higher in HBV-infected men compared to non-infected ones (10). HPV is a nonenveloped double-stranded DNA virus transmitted by sexual contact (11). Gizzo and colleagues showed that HPV infection of the sperm might be involved in decreasing the fertility rate among men through different mechanisms that may influence human embryo development (12). In a review article, Foresta and colleagues reported that the prevalence of HPV semen infection in infertile men is 10-35%; it was also shown that the motility of the sperm in the infected semen sample was lower than in uninfected semen (13).

HSV is a double-stranded DNA virus from the Herpesviridae family (14). The prevalence of this virus in semen varies from 3-50%, depending on the investigation method (15). It has been reported that there is a correlation between the presence of HSV in the semen and a decreased in sperm concentration and reduced motility (4). EBV is a ubiquitous virus that replicates in the epithelial cells and lymphocytes (16). It was found that EBV DNA was present in 40% of cases in equal frequency among normal and abnormal semen (15).

Accordingly, in this study, we aimed to investigate the relative frequency of HBV, HPV, EBV, and HSV infections and their effects on the semen quality and sperm characteristics in fertile and infertile men referred to the Ghadir Mother and Child Hospital, Shiraz, southwestern Iran.

## 2. Materials and Methods

### Study design and subjects 

In this cross-sectional study, 350 subjects were enrolled, including 200 infertile and 150 fertile men who were referred to the Ghadir Mother and Child Hospital affiliated with the Shiraz University of Medical Sciences between August to September 2016. The inclusion criteria for the infertile group was a history of infertility with failure to achieve pregnancy after at least one year of unprotected sexual contact. The exclusion criteria of the case group were disorders which affects the sperm parameters, such as azoospermia, undescended testis hydrocele, varicocele, epididymitis, and human immunodeficiency virus infection. Additionally, those who had genital lesions associated with HPV and HSV or whose spouses had histories of uterine or ovarian disorders were also excluded from the study. The fertile control group includes men who had at least one child.

Sterile containers were used for semen samples collection by way of masturbation after three days of sexual abstinence. Then semen sample quickly transported to the laboratory and stored at -20°C for further examination. The subjects were advised to wash their hands and genital area with soap and water prior to sampling.

### Semen analysis and HBV, EBV, HPV, and HSV detection

All semen samples put in incubator for 30 min at 37°C. Sperm parameters were determined according to the world health organization guidelines. Then, DNA extraction was performed using DNA extraction kit (CinaGene, Tehran, Iran) according to the manufacturer's instruction. The extracted DNA was stored at -20°C until analysis. Detection of HBV, HPV, EBV, and HSV1/2 was performed by the PCR method.

### HBV PCR conditions 

Detection of the HBV genome was done using the HBV PCR detection kit (Sinacolon, Tehran, Iran) following the manufacturer's instructions. After that, the PCR product was run on a 1.5% agarose gels.

### HPV PCR conditions

HPV detection was performed by universal GP5/6 primers. PCR reaction components were Ampliqon 2x PCR mix red (Amplicon, Denmark, Odense) and 0.4 µM of each primer in 25 µL final volume. Subsequently, amplification was done in conditions including five min initial denaturation at 95°C, 40 cycles of denaturation at 95°C for 45 sec, annealing at 55°C for 45 sec, extension at 72°C for 45 sec, and a final extension at 72°C for 10 min. The PCR products were run on a 1.5% agarose gel.

### EBV PCR conditions 

Allele ID 7 software were used to designed the sequences of primers specific for BHRF1 region of the EBV genome (Table I). Ampliqon 2x PCR mix red (Ampliqon, Denmark, Odense) plus 0.4 µM of each primer in 25 µL final volume were used for PCR reaction. Amplification was carried out as follows: initial denaturation at 95°C for 10 min, 45 cycles of denaturation at 95°C for 45 sec, annealing at 57.6°C for 45 sec, extension at 72°C for 45 sec, and a final extension at 72°C for 10 min. PCR products were then loaded onto a 2% agarose gel and visualized under UV light. The DNA extracted from the B-95 cell line (Pasteur Institute, Tehran, Iran) was used as a positive control for EBV in each run.

### HSV PCR conditions

HSV detection was performed by common HSV1- and 2-specific primers (Table I). PCR reaction components included Ampliqon 2x PCR mix red (Amplicon, Denmark, Odense) and 0.4 µM of each primer in 25 µL final volume. Amplification was carried out as follow: denaturation at 95°C for 10 min, 45 cycles of denaturation at 95°C for 45 sec, annealing at 58°C for 45 sec, extension at 72°C for 45 sec, and a final extension at 72°C for 10 min. The PCR products were run on a 1.5% agarose gel.

**Table 1:** The sequences of the primers used in the present study


**Primer**		**Oligonucleotide sequence**	**Product size**
	Forward	5'-TTTGTTACTGTGGTAGATACTAC-3'	
**HPV**	Reverse	5'-GAAAAATAAACTGTAAATCATATTC-3'	150 bp
		Forward		5'-CAGTACGGCCCCGAG TTCGTGA-3'	
**HSV**	Reverse	5'-TTGTAGTGGGCGTGGTAGAT-3'	465 bp
		Forward		5'- TACTCCTTACTATGTTGTG-3'	
**EBV**	Reverse	5'- CCTTGCCTAATATCCTAC-3'	295 bp
	HPV: Human papilloma virus, HSV: Herpes simplex virus, EBV: Epstein-Barr virus			

### Ethical considerations

The study was approved by the ethics committee of Shiraz University of Medical Sciences, Shiraz, Iran (Code: IR.SUMS.MED.REC.1397.304) and all participants signed an informed contest form before sampling.

### Statistical analysis

Data were analyzed using the IBM SPSS Statistics software version 21 (IBM Company, USA). Chi-square and Mann-Whitney tests were used to compare different parameters. P < 0.05 was considered as statistically significant.

## 3. Results

### Demographic characteristics of the participants

Of the 350 participants included in the study, 200 were infertile and 150 fertile. The mean age of the infertile group was 36 ± 7 yr, ranging from 22-61 yr. The mean age of the fertile control group was 36 ± 6.9 yr, ranging from 24-62 yr. Of the 21 semen samples that were positive for HBV, 9 (43%) were struggling with substance use disorder, but no significant association was found (p = 0.51).

### The results of the semen analysis

The mean semen volume was 3 ± 1.4 mL in the fertile group and 2.7 ± 1.6 mL in the infertile group (p = 0.59; p = 0.001). The mean sperm count was significantly lower in the infertile group compared to the fertile group (12.6 ± 15.2 vs. 54.83 ± 21.9 million/mL, respectively; p = 0.001). Sperm motility in the fertile group was 50 ± 8.4 and was significantly lower in the infertile group (26.7 ± 12.8, p = 0.001). A significant difference was seen between the two studied group in the semen volume < 1.5 mL (p = 0.006). Also, those with a sperm count of < 15 million/mL were significantly more frequent in the infertile group than in the fertile one (p = 0.001). Furthermore, sperm motility of < 32% and sperm morphology of < 4% were significantly higher in the semen of infertile men than in the fertile ones (p = 0.001). The frequency of HBV in the semen of fertile and infertile groups was not significantly associated with the sperm count, abnormal morphology, sperm motility, or semen volume.

### The results of HBV, HPV, EBV, and HSV1 and 2 detection through PCR

Our molecular results showed that 16 (8%) out of the 200 samples of infertile men and 5 (3.3%) out of 150 samples of fertile were positive for HBV, which was statistically insignificant (p = 0.06). No significant correlation was noted between the age of infertile and fertile men infected with HBV (p = 0.1). Only one sample in the fertile group was infected with HPV and done in the fertile group. Furthermore, in our study, none of the semen samples of the infertile or fertile group were positive for EBV or HSV 1 or 2.

The frequency of some bacteria including *Staphylococcus aureus, Lactobacillus, Chlamydia, Neisseria, Helicobacter, Mycoplasma, Citrobacter, Hemophilus, E. coli,* and *Klebsiella* was also detected in this sample. Coinfection of HBV with a bacterial infection occured with the following frequencies: *Staphylococcus *infection 4.8% (1 out of 21), *Lactobacillus* 4.8% (1 out of 21), *Chlamydia* 4.8% (1 out of 21), and *Neisseria* 9.5% (2 out of 21). No coinfection of HBV with *Helicobacter, Mycoplasma, Citrobacter, Hemophilus, E. coli, *or *Klebsiella* was found (17).

## 4. Discussion

Infertility has become a major health problem and male factors are responsible for up to 50% of the cases (18). The causes of infertility in a large number of infertile men are still unknown, but it seems that urogenital infections are responsible for infertility in 6-10% of cases (19, 20). Several studies have shown that viral infections including HBV, HPV, EBV, and HSV can infect semen and cause infertility by influencing sperm characteristics and semen quality (3, 4, 5).

It has been reported that progressive motility and percentage of normal sperm morphology in infertile men infected with were significantly lower in comparison with HPV-negative cases (21). Also, a significant association of HSV infection with a lower seminal volume and a lower mean sperm count was reported (22). Moreover, another study showed that sperm motility and normal sperm morphology were significantly negatively affected in HBV-positive men (23).

The results showed that 8% and 3.3% samples of infertile and fertile men were positive for HBV, respectively, which was not statistically significant. Moreover, only one sample of the fertile men was positive for HPV. Furthermore, none of the semen samples of the infertile or fertile groups was positive for the presence of EBV or HSV1/ 2.

Although more semen samples of our infertile participants were positive for HBV than of the fertile group, the difference was not statistically significant. In agreement with our study, Zangeneh and colleagues reported that the frequency of HBV in infertile persons who they studied was very low and was not statistically different from fertile men (24). A study in Ahvaz also showed a very low frequency of HBsAg among infertile couples (25).

Moreover, it has been reported that none of the semen samples of infertile men who they examined were positive for HBV DNA and the mentioned that the low prevalence of HBV infection in their population might have been the cause of the negative results (26). On the other hand, a case-control study that compared men with HBV infection to those without HBV showed an increased risk of infertility in HBV-infected men (8). Also, in a systematic review, it was stated that HBV infection could cause male infertility (4). Therefore, according to the mentioned studies, it seems that the low prevalence of HBV infection in infertile persons, as well as the small sample size of our study groups, may have influenced the HBV association with male infertility.

In the case of HPV, only one of the fertile group participants and none of the infertile participants were positive for HPV. In this regard, Bezold and colleagues reported that 4.5% of the semen samples of infertile men who they studied were infected with HPV (26). In contrast, some studies have reported a significantly higher prevalence of HPV in infertile groups and have also shown a significantly lower sperm motility and count in HPV-infected semen (27-30). Moreover, a systematic review mentioned that HPV infection might cause male infertility, but available data are conflicting (4). Furthermore, a meta-analysis by Lyu et al. showed a twofold increased risk of infertility in men with HPV-infected semen (31).

Semen samples of both fertile and infertile participants were negative for EBV. In agreement, Bezold and colleagues reported that EBV was detected in only one of the samples they examined (26). A study conducted by Neofytou and colleagues showed that although EBV was present in 45% and 39.1% of the semen samples of their fertile and infertile participants, respectively, it was not significantly associated with infertility (15). Kapranos and colleagues also showed that 16.8% of their study's semen samples of infertile people were infected with EBV, but there was no association between EBV infection and abnormal sperm motility or semen count (3). Therefore, considering the aforementioned results of the previous studies, it seems that EBV does not have a major role in infertility.

Semen samples of both fertile and infertile participants were negative for HSV1 and HSV2. In line with our results, HSV1 was present in 2.1% and 2.5% of the semen samples of infertile and fertile subjects, respectively, which was again not statistically significant (15). However, the results of a study performed by Monavari and colleagues showed that 20 and 15% of their infertile participants were infected with HSV1 and 2, respectively (32). Moreover, 56.6% of semen samples of infertile people were infected with HSV1. They also showed that the semen samples infected with HSV1 had significantly lower sperm motility than the non-infected one (3). Moreover, HSV was present in 3.7% of the semen samples of the infertile men and HSV presence was associated with a decrease in sperm count and motility (26). Furthermore, in a review by Ochsendorf et al., a significant relationship was observed between the presence of HSV and infertility (33). According to these studies, it seems that HSV might be involved in infertility at least in some areas, which might be related to a high frequency of HSV infection in those populations.

The relatively small sample size was a limitation of our study. Using the conventional PCR method and not real-time PCR can be considered as another limitation of this study.

In sum, while a number of studies have shown associations between infertility and HBV, HPV, and HSV, some others do not support these findings. This strong discrepancy may partly come from differences in factors including the sample size, different geographical distribution of the viruses, and lifestyle (sexual behavior) of the studied subjects. Moreover, technical issues including the sensitivity of detection methods (PCR or real-time PCR) as well as differences in the extraction procedures may also explain discrepancies among different studies.

## 5. Conclusion

According to our results, among the viruses investigated in this study, only HBV and HPV were detected in the semen samples; however, their frequency revealed no significant difference between infertile and fertile groups. The frequency of HBV was not significantly associated with the sperm count, abnormal morphology, sperm motility, or semen volume in either study group.

##  Conflict of Interest

All authors declare no conflict of interest.
